# When inequality looms large: the cumulative impacts of discrimination on sexual minorities’ late life experiences

**DOI:** 10.3389/fpubh.2026.1941078

**Published:** 2026-10-16

**Authors:** Lisa R. Miller

**Affiliations:** Eckerd College, St. Petersburg, FL, United States

**Keywords:** cumulative (dis)advantage, end-of life, LGBTQ health, sexuality, successful aging

## Abstract

**Introduction:**

Research suggests that older sexual minorities face distinctive challenges in late adulthood compared to their heterosexual counterparts. However, attention to the role that lifelong discrimination plays in the aging experiences of older sexual minorities has been insufficient.

**Methods:**

Utilizing life story interview data with older (55+) sexual minorities, I examine how lifelong exposure to structural inequality contributes to aging-related challenges and the development of unique assets in late adulthood.

**Results:**

The results suggest that older sexual minorities face three distinct challenges in late adulthood: economic marginalization, medical distrust and subsequent rejection of medical care in long-term care facilities, and suicidal thoughts and intent. In addition, participants possessed unique assets for confronting aging-related challenges, namely community-centered care approaches.

**Discussion:**

Taken together, the results suggest that much greater attention should be paid to cumulative inequality processes in the lives of older sexual minorities, including how this population can avoid the accumulation of disadvantage over time.

## Introduction

There are currently close to 3 million lesbian, gay, bisexual, transgender, and queer (LGBTQ) adults over the age of 50 in the United States today, and this figure is projected to increase to 5 million by 2030 (1). It is, perhaps, then not surprising that the older sexual minority (SM) population has received increased scholarly attention in recent years, although this distinctive group remained invisible in the larger field of gerontology for many decades (1). Indeed, research consistently reveals that older sexual minority adults face numerous distinctive challenges compared to their heterosexual counterparts, including barriers in accessing informal and formal care, health disparities, and social isolation (2–5). Despite the fact that the study of LGBTQ aging is bourgeoning, researchers have not paid sufficient theoretical and empirical attention to how exposure to early life discrimination may impact the quality of life and wellbeing of older sexual minorities. Accordingly, in this article, I ask how exposure to early life stressors may contribute to older lesbian, gay, bisexual, and queer (LGBQ) people facing distinctive challenges and developing assets in late adulthood.

Cumulative inequality approaches are fairly common in the study of aging and the life course, along with health disparities research. Research consistently shows that exposure to stressful early life events (e.g., prejudice, discrimination, violence, poverty, abuse) may lead to a worsened quality of life for individuals in midlife and late adulthood (6, 7). In other words, the effects of stressful events are rarely confined to the life course stage wherein the events occur; rather, the negative repercussions of discrimination accumulate over time, often morphing into large-scale consequences for older individuals (8). Yet, such a perspective is rarely integrated into the study of LGBTQ+ aging.

Using originally collected life story interview with 60 older (55+) LGBQ individuals, I ask how exposure to discrimination earlier in life may cascade and compound, leading to older sexual minorities facing heightened aging-related challenges. In doing so, I examine how barriers to successful aging in late adulthood may be driven by past discriminatory experiences. Simultaneously, I ask whether older sexual minorities may develop unique assets that help them confront barriers to positive wellbeing in late adulthood and, in doing so, argue that the study of LGBTQ+ aging needs to move beyond a deficit perspective.

## Background

### Cumulative inequality processes

It is well known in the broader study of social inequality that systematic disadvantages compound over the life course. Indeed, cumulative inequality theories advance that exposure to early-life disadvantages (e.g., abuse, prejudice, discrimination, poverty, violence) increase exposure to additional risk over time, including poor health later in life (6, 7, 9). Key to this approach is an understanding that exposure to stressful early life events—including discrimination—is consequential for later life trajectories (10). For example, as Hatch explicates, discrimination can “occur in a layering effect with one hardship remaining present and the next building on top of it in a cascading sequence” (8, p. 3). In other words, discriminatory experiences accumulate over time, resulting in smaller effects of discrimination manifesting into much larger consequences for individuals later in life. There is also considerable evidence for the theory, as researchers consistently find that socioeconomic and racial inequalities lead to heightened health disparities later in life (7, 11–13). Yet, this theoretical perspective is rarely integrated into the study of LGBTQ+ aging, although life course analyses of older LGBQ adults acknowledge that past stressful events have measurable impacts on health outcomes among this population (14–16).

Only a small handful of scholars in the field of LGBTQ+ studies have conceptualized, theorized, or empirically studied how cumulative inequality processes unfold among sexual minority populations. Multiple review articles outline future research directions for studies of cumulative inequality among LGBTQ+ populations, highlighting how exposure to discrimination may be consequential for quality-of-life indicators (e.g., financial wellbeing, physical & mental health, social connections) in late adulthood (16, 17). Similarly, life course scholars emphasize the important role of historical context, noting that the aging and life course trajectories of boomers (born between 1946 and 1964) and the silent generation (born between 1928 and 1945) may also differ; these differences are likely due to coming of age during varying historical periods (2, 16). Further, a study by Fredriksen-Goldsen et al. uses a life course perspective to document that negative life events and transitions influence health and wellbeing later in life (14). Consistent with this, other scholars assert that exposure to early life discrimination may be consequential for the economic stability of same-sex led households. For example, it is well documented that same-sex couples have been denied access to federal programs (e.g., social security, Medicaid, retirement planning, and retiree health insurance plans), which likely leads to poor economic outcomes for older LGBTQ+ individuals (18). For example, same-sex couples, on average, earn 34.7% less in retirement income and social security payments (19). Similarly, exposure to discrimination may lead to delayed retirement, as 25% of LGBTQ+ who are 65+ still work—a statistic that is 18% higher than the general population (20).

Consistent with the principle of cumulative inequality, social identity approaches to health demonstrate the linkage between discrimination and wellbeing among minoritized populations, including sexual minorities. It is widely accepted that lower social status consistently predicts declines in health (21, 22). Similarly, minority stress theories posit that exposure to stressors in social environments (e.g., prejudice, discrimination, and health) is linked with poor health outcomes among sexual minority individuals (23, 24). Both theoretical approaches implicitly assume that discrimination may shape health outcomes later in life, but additional research is needed to understand how early life risks shape the aging and life course trajectories of older sexual minorities (14, 15, 25, 26). Relatedly, little minority stress research focuses specifically focus on the impacts of minority stress on the health and wellbeing of the older sexual minority population (27, 28). More generally, there a lack of retrospective, longitudinal, and/or qualitative data that allows researchers to fully understand how cumulative inequality *processes* unfold over time among sexual minorities (14, 26, 29).

### Barriers to successful aging among older sexual minorities

It is possible that older sexual minorities face distinctive aging-related challenges, which may pose barriers to successful aging: the ability to maintain a high quality of life and health in late adulthood (30–32). For example, it is well documented that older sexual minorities face distinctive challenges (e.g., caregiving, health-related, and social isolation) in late adulthood. Due to systematic exclusion from the institutions of marriage and family, older sexual minorities may have fractured relationships with families of origin, be single, and also be without children of their own (14, 33, 34). Unlike many heterosexual individuals who rely on children and extended family for care, many older sexual minorities may be without access to an informal caregiver. Similarly, it is not uncommon for older sexual minorities to express apprehension about staying in long-term care settings, often due to fears of identity concealment in order to avoid homophobic discrimination (5, 33, 35). Further, heightened exposure to poverty and economic stability in late adulthood may leave older sexual minorities without the resources necessary to access informal and formal care alike (26, 33). Thus, older sexual minorities regularly experience heightened caregiving challenges compared to their heterosexual counterparts.

Relatedly, there is evidence that older sexual minorities experience unique advance-care-planning challenges, including a lack of financial resources to complete legal paperwork (36). In fact, one study suggests that fewer than half of all middle-aged sexual minorities have a living will or power-of-attorney (37). Simultaneously, older sexual and gender minority (SGM) individuals often understand the value of formal care planning, as they see it as a vital way to have their wishes honored (38, 39).

Further, older SM populations commonly experience health disparities compared to their heterosexual counterparts. It has been documented that older sexual minorities have higher risk of depressive symptoms, suicidal thoughts, drug/alcohol abuse, and challenges with accessing healthcare (3, 40). There is also preliminary evidence that this population experiences higher rates of cardiovascular disease (41), chronic pain (42), sleep problems (43), and higher cognitive impairment (44–46). Further, there is some evidence that exposure to minority stress (e.g., prejudice, discrimination, violence) is associated with poor health among older sexual minorities (15, 29, 47). However, because these studies are largely quantitative and focus on statistical correlation at one point in time in terms of the data collection process, they do not offer an in-depth understanding of the aging and life course trajectories of older sexual minorities.

Older sexual minorities also commonly report higher rates of social isolation, often explained by family estrangement, singlehood, living alone, and being child free (48, 49). Findings of social isolation are particularly concerning, as this phenomenon is linked with worse mental health outcomes (48) and inadequate utilization of health and social services in times of need (50). Many of the aforementioned risk factors for social isolation—particularly family estrangement, being child free, and instability in housing—are also likely linked with exposure to discrimination across the life course.

Taken together, the distinctive aging-related challenges (e.g., caregiving, advanced-care planning, health-related, social isolation) faced by older sexual minorities indirectly offers some evidence for cumulative inequality perspectives. Although the role of exposure to early life stress is often acknowledged in prior studies, it is rarely centered in empirical investigations of older sexual minority populations. Thus, the current study fills this key gap in the literature, specifically utilizing retrospective data to document how exposure to workplace and medical discrimination impact the aging and health trajectories of older sexual minorities. In doing so, the article also advances understandings of how exposure to early life stressors—including discrimination—may interfere with older sexual minorities’ ability to successfully age.

### The development of unique assets and coping mechanisms

Simultaneously, it is important to consider older sexual minorities’ capacity for resilient aging: the ability to adapt to aging-related challenges (51). Despite exposure to minority stress across the life course, it is possible that older sexual minorities develop unique coping mechanisms to deal with emerging challenges. Indeed, the “crisis competence” perspective in the study of LGBTQ+ aging emphasizes that exposure to homophobic discrimination over time leads to sexual minorities being able to better cope with aging-related challenges (4, 52, 53). Empirical evidence also confirms this reality; according to the MetLife Mature Market Institute, 75% of SGM respondents report that their sexual and gender minority (SGM) status has prepared them well for aging (54). Despite older sexual minorities’ capacity for resilient aging, studies on LGBTQ+ aging overwhelmingly examine disadvantages that older SGM populations face.

Moreover, in the study of marginalized populations more generally, there remains a tendency to overly emphasize a deficit perspective; scholars overwhelmingly examine violence, discrimination, and oppression in the lives of vulnerable groups, resulting in stories about the strengths that these individuals possess going untold (55). Because the study of LGBTQ+ aging also suffers from this problem, there remains an urgent need for scholars to consider potential positive outcomes of older sexual minorities’ exposure to marginalization over time (56). Moreover, some attention has also been paid to how the activation of group-level strengths allows some marginalized individuals to avoid the accumulation of disadvantages over time (57, 58). Social identity approaches to health similarly find evidence for a “social cure” hypothesis, wherein collectivist approaches among minoritized groups lead to the activation of resources that can offset negative consequences of discrimination (21, 22, 59). However, questions regarding pathways toward resiliency—including creative strategies for confronting challenges to positive wellbeing in late adulthood—merit much greater attention in research on SM aging.

Further, there is some preliminary evidence that older sexual minorities may experience some advantages in the aging process. Despite facing isolation, LGBTQ+ individuals are more likely to turn to friends for social support—which may lead to positive health outcomes (1). Similarly, LGBTQ+ people may adopt more of a collective mindset in late adulthood, resulting in the sharing of resources to care for one another (33). Further, prior studies find that same-sex couples are more likely than heterosexual couples to utilize egalitarian approaches when caring for aging parents, engaging in advance care planning, and managing medical care for spouses (1, 16, 60).

### The current study

Drawing on life story interview data with 60 older (55+) LGBQ individuals, I take up two key aims in this article: (1) To shed light on how lifelong exposure to structural inequality shapes older LGBQ people’s quality of life and wellbeing in late adulthood. (2) To document unique community assets and coping strategies that older sexual minorities possess in late adulthood. In doing so, the current study elucidates how discrimatory experiences faced earlier in life complicates one’s ability to successfully age. Simultaneously, the current study avoids the sole use of a deficit approach commonly found in the study of LGBTQ+ aging, namely by considering distinctive assets and coping mechanisms that older sexual minorities possess to confront aging-related challenges.

## Materials and methods

In this article, I utilize life story interview data with 60 older (55+) lesbian, gay, bisexual, and queer people who reside in the United States. The life story interview method is particularly ideal for documenting how people’s lives unfold over time, and it also allows researchers to gain insight into how historical context shapes people’s lives (61). Life story interviews have been used by other scholars as well to study the lives of older sexual minorities. For example, as noted by Ramirez Vallez, the logic of the life story interview method is “based on the queer notion of memory as a counteractivity to dismembering” (47, 62). In other words, life story interview methods allow researchers to remember the unfolding of older LGBQ lives during moments of political and cultural erasure. Further, this method is ideal for documenting cumulative inequality *processes* over time, due to its analytic attention to how major life events and turning points shape later life experiences.

In the life story interviews, I use a semi-structured interview guide, which began with participants being asked background questions about their families, schools, and hometowns; these “low stakes” types of questions were asked to build rapport and trust before seeking to solicit more private and sensitive information. Thereafter, I ask participants a range of questions about “coming out,” marriage, family, the workplace, health, and aging. Probing techniques—using clarifying questions—were also utilized when participants’ responses were too brief. Although I ask about a wide variety of topics, the current manuscript centrally relies on any and all data that sheds light on how risks faced earlier in life shape aging experiences, including inequitable outcomes and capacity for resiliency in late adulthood. On average, interviews lasted 1.5 h, were audio-recorded with permission, and were transcribed verbatim; pseudonyms were also assigned to all study participants.

To be eligible for the study, participants had to be 55 or older, identify as a sexual minority (e.g., lesbian, gay, bisexual, queer), and reside in the Southern or Midwestern regions of the United States; I focus on these particular regions because they have received far less attention than others in social scientific studies on the LGBTQ+ population (63). The study includes two key generations: boomers (born between 1946 and 1964) and the silent generation (born between 1928 and 1945). The latter lived through eras characterized by turbulent homophobia, including the McCarthy era wherein a literal witch-hunt against lesbian, gay, and bisexual government employees unfolded (2). The criminalization and pathologization of homosexuality was also pervasive during the period in which the Stonewall generation came of age. In contrast, the Boomer generation came of age during the Stonewall Riots, a time when LGBTQ+ people experienced increased political and cultural visibility as a result of a fight for political rights. Consequently, there may be marked differences in the life course experiences of boomer and the silent generation, an issue I briefly return to throughout the results section when relevant.

Due to the fact that older sexual minorities are a hard-to-reach population, I rely on non-probability sampling methods (e.g., convenience, snowball). Most existing studies of this population similarly use non-probability sampling methods (14). I identified key gatekeepers at community-based organizations (e.g., LGBTQ centers, senior centers, assisted living facilities) that serve older LGBTQ+ populations, who, in turn, played a pivotal role in helping me recruit for the study. In addition, the study was advertised by hanging flyers containing study information at various locations (e.g., coffee shops, bars, houses of worship, senior centers) and was shared with varying online communities for SGM populations.

Data were coded with ATLAS.ti, a qualitative software program. Consistent with the life story interview approach, I sought to identify major life events, transitions (e.g., a significant event resulting in a change in one’s role or identity), and turning points (e.g., life altering events that change the course of one’s sexual identity trajectory) that altered late life experiences (61). In doing so, I also sought to document how participants made sense of their aging experiences, consistent with qualitative analytic approaches that prioritize uncovering meaning-making processes. Further, detailed notes were taken during and after all interviews, resulting in me entering the coding process with ideas about key themes present across interviews. I also used *line-by-line* coding to identify new patterns in the data that I was not yet aware of and then utilized *focused coding* to merge some of the overlapping themes into more general categories (64, 65). Consistent with qualitative analysis, I also wrote integrative memos in order to record reflective thoughts about themes that were emerging in the data (65).

### Statement of positionality

Insider/outsider dynamics inevitably shaped the collection of the data. As a lesbian woman, I was able to build trust and rapport with participants through sharing my identity status before or during interviews. Simultaneously, as a millennial woman, I did not belong to the same cohort of my participants, resulting in me exercising caution in assuming I could occupy the full status of an expert when interviewing participants. Consequently, I often relied on a more open-ended interviewing approach, allowing participants to play a vital role in the direction of the interview in telling their own stories. Indeed, it became apparent early in the interviewing process that I did not always know the correct questions to ask as a younger lesbian woman, due to sometimes making erroneous assumptions about what it meant to live as a sexual minority person in earlier historical periods. Consequently, interviewing was a reflective process insofar as I continuously worked to ensure that I was fully capturing a trustworthy and accurate account of participants’ stories; in doing so, I sought to honor the view of the social actor (66).

## Results

### Key themes

In what follows, I document cumulative inequality processes in the lives of older sexual minorities, highlighting how risks faced earlier in life often introduce new and continual challenges in late adulthood. As displayed in table one, the study has four major themes: (1) Economic stressors experienced earlier in life contribute to heightened financial challenges in late adulthood. (2) Due to exposure to medical discrimination and abuse earlier in life, participants report a deep sense of distrust of medical staff in formal care settings. (3) Participants report a desire to pursue assisted suicide in lieu of the utilization of medical services in long-term care settings. (4) A coping mechanism for aging-related challenges was the adoption of community-centered care, wherein participants assumed responsibility for tasks associated with daily living and medical maintenance for other SGM individuals. Below, I further elucidate these key research findings (see Table 1).

**Table 1:** Key study themes.

	Definition and context
Challenges	
Economic marginalization	Heightened risk for poverty (via inadequate savings, failure to acquire wealth, and a shortage of waves), due to exposure to workplace discrimination across the life course.
Medical mistrust	A reluctance to seek sustained medical attention in formal care settings, which may be linked with exposure to medical discrimination across the life course.
Suicidal ideation and plans for assisted suicide	A desire to pursue assisted suicide—coupled with suicidal thoughts—as an end-of-life plan when one can no longer care for her/himself. Often related to fear of abuse in medical settings.
Assets	
Community centered care	A desire to share in the management of everyday tasks and routines (e.g., meal prep, cleaning, transportation), medical management and assistance, and the provisioning of social support with other members of the LGBTQ+ community.

### Economic marginalization

In this section, I ask how exposure to homophobic stressors in the workforce contributes to heightened aging-related challenges in late adulthood, namely additional financial distress. Although poverty is commonly reported as a challenge among older adults, it is potentially exacerbated by lifetime exposure to homophobic discrimination (26, 33). Job discrimination motivated by homophobia took varying forms among participants, including refusal of hire, wrongful termination, and poor performance reviews. Consequently, participants often underscored long-term financial consequences of exposure to lifelong discrimination, including heightened poverty and inability to secure affordable housing in late adulthood. Below, I describe how economic marginalization manifested in the lives of participants in late adulthood.

Indeed, participants’ narratives underscore that early life disadvantages via job discrimination was often associated with heightened financial precarity in late adulthood for sexual minorities. For example, Steve, an 80-year-old gay identified married man, experienced much homophobic rejection earlier in life. Steve notes that he was falsely accused of indecent exposure during his twenties, which he believed was motivated by homophobic bias. He has also wondered if the subsequent mark on his legal record later contributed to him being denied a job. Further, he notes that such discrimination has taken a toll on many sexual minorities’ ability to maintain financial stability later in life:

Gay people have never had the advancements in jobs and all of that because of who they were…They have not built IRAs. We never thought about it until we were in our late 40s…We know people that are just going by the skin of their teeth. They’re on food stamps, and their job never paid enough for them to accrue anything for retirement. And their social security certainly is not good.

Here, Steve elucidates the nature of job discrimination from earlier in life, which he acknowledges can lead to poverty in late adulthood.

Other participants also echo Steve, noting challenges in the ability to accumulate savings to properly prepare for the retirement years. Joseph, 83-year-old married gay man, remarks:

Due to a lack of promotions and bonuses, saving was not something they [gays, lesbians, and bisexuals] could do because they could hardly live on just what they were getting. So, they enter a timeframe in their lives where they do not have the finances…

Joseph’s remarks highlight the possibility that homophobic discrimination contributes to a loss of wages over time, impacting one’s savings and acquisition of wealth. Notably, participants’ narratives are consistent with prior studies that similarly find that sexual minorities experience disparities in pay across the life course (1).

Importantly, there may be variation in exposure to economic marginalization in late adulthood. For example, there is some evidence that baby boomers experienced more job discrimination than the silent generation, due to higher rates of identity disclosure that exposed them to homophobic rejection in the workplace (33). Consequently, baby boomers’ experiences with financial instability in late adulthood may be worse than older cohorts. In addition, there may be important gender differences in exposure to financial distress in late adulthood. For example, lesbian and bisexual women are likely to face higher rates of poverty in late adulthood, due to exposure to long-term wage loss over time via the gender wage gap (14, 26).

### Medical mistrust

Next, I examine the long-term consequences of homophobic stressors in medical settings, namely medical mistrust (e.g., a lack of confidence in medical providers). Participants commonly reported fears of discrimination from medical providers and staff in long-term care settings. Particularly alarming is that the development of medical mistrust over time often contributed to participants’ rejection of the idea of receiving healthcare in institutionalized care settings, a decision that may exacerbate underlying health conditions.

Participants often reported earlier life instances of medical discrimination, which they perceived as having long-lasting effects. For example, Connie (68, marred, lesbian woman) recalls unequal treatments from medical doctors, which shaped her plans for care in late adulthood. Consequently, she remarks:

I know that people with AIDS, guys with AIDS have gone through absolute hell having to live in these facilities. They’ve been beaten up by the nurses. I mean, it’s just atrocious…there are too many older women being raped.

As a consequence of remembering medical mistreatment of her gay male brethren, Connie had inherited a sense of generational trauma. Further, Connie expressed concern about sexual assault in formal care facilities, a fear that was driven by the potential for both sexist and homophobic mistreatment. Consequently, she elaborated that she intends to have her sister provide care for her when she is no longer able to do so.

Others echoed Connie’s concerns, expressing fears of ageist and homophobic mistreatment in formal care settings. Such fears typically entailed concerns about medical neglect at the hands of staff, including concerns that staff would not properly attend to hygiene, fail to treat medical conditions, or inflict physical abuse. For example, Patricia (64, married, lesbian) notes:

How would we be treated by the staff? Would we be treated differently than the other people would?

Patricia’s fears emphasize the reality of denial of medical care and/or subpar medical treatment. Further, participants had widespread fears about having to “re-enter the closet” as a tactic to avoid discrimination from medical providers in long-term care facilities, which often led them to reject the idea of staying in a formal care setting in late adulthood.

Given the historical legacy of medical discrimination faced by LGBTQ+ adults, it is unsurprising that numerous participants had developed medical mistrust. Indeed, generational trauma was often the source of mistrust of staff in paid care settings, as participants commonly remembered medical mistreatment during earlier historical periods. Here, we see that the effects of past social inequality persist and have the potential to widen health disparities faced by older sexual minorities documented in prior studies (41, 45). Not unlike job discrimination, prior instances of medical discrimination did not solely impact sexual minorities’ experiences during the life course stage in which they occurred.

### Suicidal ideation and plans for assisted suicide

Participants’ narratives also revealed that suicidal thoughts or plans were not uncommon. Prior research similarly finds that older SGM adults are at elevated risk for suicide compared to their cisgender and heterosexual counterparts (67, 68). Although adolescent and young adults in the LGBTQ+ community are often assumed to be at risk for higher suicidal ideation and completion, it quite rare for public health scholars and professionals to consider how and whether older SGM individuals are similarly at risk. Stigmatization, rejection, and isolation are documented as key correlates of higher suicidal thoughts and completion among LGBTQ individuals, more broadly (25). Although such risk factors may play a role in suicide among older LGBTQ+ adults, other factors are also likely responsible—including ageist and homophobic fears of neglect and abuse in late adulthood. Below, I detail how suicide emerged as an end-of-life care plan when participants could no longer care for themselves.

As previously mentioned, participants’ narratives revealed that they were deeply fearful of medical neglect in formal care settings. Consequently, many participants intended to avoid such medical mistreatment at all costs, including possible suicide completion. For example, Gloria (72, married, lesbian) discussed at great length what her end-of-life care plans are, noting that she plans to pursue suicide before being willing to enter a paid care facility. She remarks,

I’m not going to be out of it in a nursing home where you’d be drugged even more and maybe restrained and people not change you. I love life, but that’s not the life I want. That’s because they can extend life beyond what’s quality of life. And I do not believe in that.

Gloria’s comments highlight that medical mistreatment—likely as a consequence of ageist and homophobic abuse—would severely compromise her quality of life. Accordingly, her narrative—not unlike many other participants—suggests that she deeply desires a dignified life and death, and that suicide is more likely to produce that outcome.

Other participants spoke of the importance of bodily autonomy in late life, suggesting that suicide might help them achieve this. For example, Norman (71, single, gay-identified man) remarks:

If I got to the point where my life was not … enjoyable … if I got bedridden or something like that I’m ready to go. I do not want to live like that. I do not want to live in a bed.…I would probably want to end my life myself.

Much like Gloria, he understands that aging can produce debilitating effects on the body, and he does not see a life where he has lost control of bodily functions worth living. The recognition of bodily autonomy as a key value in late adulthood may also be a product of earlier exposure to discrimination, wherein one’s ability to make agentic bodily and sexual decisions was severely constrained.

Here, additional reflection regarding cohort differences in the experience with suicidal planning is in order. Specifically, a desire for assisted suicide in late adulthood was more commonly reported by baby boomers than the silent generation, likely due to the fact that the former generation may have been more intensely impacted by medical discrimination during the HIV-epidemic. Indeed, evidence suggests that baby boomers experienced the effects of the HIV epidemic at higher rates than the silent generation, due to higher probability of being sexually active in their late teens through the 40s during this period (69).

### Community centered care

Lastly, I consider unique assets and coping mechanisms that older adults possess, potentially as a consequence of lifelong exposure to structural inequality. I find evidence that older sexual minority adults embrace community-centered care, departing from their heterosexual counterparts who often try to maintain independence as long as possible. Many participants also did not have children who could care for them in late adulthood, and thus visions of alternative care arrangements in late adulthood often arose out of necessity (33). Accordingly, I argue that communal care approaches also serve as a unique coping strategy for adapting to the aging process. Below, I visit Joan’s and Barbara’s stories to elucidate visions of community-centered care that were common among participants.

There is a long history in the queer community of LGBTQ+ people taking care of one another, a phenomenon that dates back to the HIV epidemic (34); given this historical precedent, it is perhaps unsurprising that participants reported strong desires to age “in community” with other SGM individuals, as they saw social support in late adulthood as crucial for successful aging. For example, Joan (66, married, lesbian) notes:

Before we had to take care of each other. We had to stay together because no one else would…. I know of many, many cases where the friends have come together, provided food, provided transportation, and been by the bed when somebody dies.

Joan’s commentary reveals that SM people experienced considerable familial rejection and discrimination in medical settings. Due to widespread discrimination, LGBTQ+ people have long understood the necessity of chosen family and cultivating sources of social support (34). Thus, Joan—and many participants like her—planned to share in caregiving responsibilities, including meal preparation, transportation to/from medical appointments, the meeting of basic needs, and even medical assistance when a friend is on their death bed. Notably, some gender differences did emerge insofar as lesbian participants were more likely than gay male participants to envision assisting with caregiving and medical-related tasks.

However, regardless of gender, many participants envisioned shared housing units and land as a key feature of community-centered care. Barbara (61, married, lesbian) narrates:

What I’d like to do is have a large community…where you had a big house, the central house, and then everybody had their little small homes out around it, so an intentional community…. You could have a shared dinner, meals if you chose to, or you could go out at your place…. If you are in trouble, you can call somebody and they are there.

Here, Barbara highlights the fact that the maintenance of a home and property would involve shared duties, but that co-housing would be a key source of social support in late adulthood. Barbara elaborates on the importance for communal living, mentioning that she even sees herself sharing land with other sexual minority women. Others shared Barbara’s vision, often mentioning that living in LGBTQ+ exclusive intentional living communities would also shield them from potential discrimination from their heterosexual counterparts. Older lesbians, in particular, dreamed of shared gardens and towing the land together. However, sexual minority men also dreamed of a “cohousing revolution” in America, where they could “age together” with other LGBTQ+ people; their vision was also meant to combat loneliness and social isolation among sexual minority communities.

## Discussion and conclusion

In this article, I investigate the challenges faced and assets possessed by older LGBQ individuals. In doing so, I considered the role of cumulative inequality processes in the lives of older sexual minorities. The results revealed that lifelong exposure to job discrimination and financial precarity was perceived as associated with economic marginalization in late adulthood among participants. Further, according to participants, exposure to medical discrimination across the life course led to medical distrust (e.g., reluctance to seek sustained medical attention in formal care settings) among their community in late adulthood. Similarly, a fear of abuse in medical facilities contributed to participants’ expressions of suicidal thoughts and intent. Finally, participants also possessed a unique asset that prepared them for effectively coping with the challenges of aging: community-centered care.

The findings underscore the importance of the study of cumulative inequality in the lives of older sexual minorities. It is well understood in life course approaches to inequality that the effects of stressful life events (e.g., prejudice, discrimination, violence) compound and may worsen quality of life in late adulthood (6, 7). Consistent with this perspective, the effects of lifetime exposure to discrimination in the job market often contributed to heightened poverty and economic marginalization in late adulthood. Indeed, economists have similarly found that job discrimination may result in a loss of wages earlier in life, leading to fewer retirement savings, less wealth acquisition over time, delayed retirement, and greater difficulty paying off one’s mortgage (18, 70). Similarly, participants’ exposure to medical discrimination earlier in life was linked with disastrous effects later in life, namely medical distrust, reluctance to seek medical care in long-term care facilities, and suicidal ideation and planning. Consequently, evidence emerged that the effects of early life discrimination were not confined to the life course stage where the event took place; rather, such effects are rarely discrete or isolated (8). Over time, the effects of homophobic discrimination generally widened, creating heightened aging-related challenges for participants. Consistent with broader critiques of the concept of successful aging (71), older sexual minorities—not unlike other minoritized groups—may also lack the resources to optimize their financial, mental, and physical health in late adulthood as a result of exposure to discrimination in the workforce and medical establishment.

Nevertheless, participants display great propensity for resiliency in late adulthood, suggesting that it may be possible for marginalized groups to utilize assets and strengths to combat cumulative disadvantages. Consistent with prior research on the crisis competence perspective (4, 52), older sexual minorities developed unique coping mechanisms that helped them age successfully as a result of exposure to inequality over time. In particular, participants embraced community-centered care, often reporting plans to share in the management of everyday tasks and routines (e.g., meal prep, cleaning, transportation), along with medical management and assistance. The possibility of communal living also offered promise that older LGBQ people would secure social support, which is pivotal for combatting social isolation, loneliness, depression, and challenges with caregiving that are commonly found among this population in late adulthood (33, 72). Further, community-centered approaches to aging may also protect participants from facing homophobic discrimination in paid care settings that are predominately heterosexual (33). Other research similarly finds that older sexual minorities adults learn valuable lessons through resources provided by LGBTQ+ communities, including insights about residential choices, recommendations for medical and service providers, and care decision-making free of gender roles (56, 60). Future research should similarly depart from deficit approaches to the study of LGBTQ+ aging by further considering what other assets that older LGBTQ+ individuals possess that may allow them to more easily adapt to end-of-life challenges that arise.

Further, older LGBQ people’s visions of aging in late adulthood often departed from heteronormative (heterosexual-centric) approaches that predominately rely on biological family for care. Indeed, heterosexual individuals often embrace the ability to maintain independence as long as possible, often expressing preferences to “age in place” (33, 73). In contrast, older sexual minorities embraced an interdependent approach to care, wherein they envisioned relying on one another to confront challenges that arise in the aging process; this phenomenon is likely driven by the fact that reliance on chosen families has long been an intrinsic part of queer history and culture. Moreover, consistent with prior research that suggests older sexual minorities disrupt heteronormative understandings of successful aging (74), participants also challenged the view that successful aging requires the maintenance of independence, namely through adopting interdependent approaches to care.

The findings also suggest that exposure to collective trauma has lasting impacts on communities. Indeed, prior research shows that collective trauma is linked with higher rates of psychological distress, health-harming behaviors, and coping mechanisms (75, 76). In this study, participants’ stories reveal that the HIV epidemic operated as a collective trauma, often leaving a lasting impact upon how participants navigated medical and end-of-life care decision making processes. In particular, participants noted that exposure to medical neglect during the HIV epidemic contributed to their reluctance to stay in long-term care facilities, fearing that they would not receive equitable care from medical providers; consistent with this, prior studies show that medical distrust as a consequence of discrimination is often linked with lower rates of healthcare utilization (including the decision to skip preventive health screenings), unmet medical needs, inadequate medical supervision, missed diagnoses, and potentially worse health outcomes (33, 75, 77, 78). Notably, even individuals who have not experienced medical trauma first-hand—such as lesbian and bisexual women—may develop medical mistrust by virtue of hearing about negligent medical encounters from other community members (75). Further, due to medical mistrust in the healthcare system, participants often reported that they would opt for suicide as an end-of-life plan when they could no longer care for themselves, consistent with prior research that shows that collective trauma is linked with health-harming behaviors (76). It is important for researchers to continue to examine how medical mistrust impacts the mental and physical health outcomes of older LGBTQ+ people who decide to forgo medical care in paid care settings.

Simultaneously, exposure to collective trauma can facilitate the development of creative strategies that lead to resiliency (59, 76). Indeed, participants reported that—due to a long history of caring for one during the HIV-epidemic as a consequence of medical neglect—they embraced community centered care. The adoption of shared care served as a key coping mechanism and, in turn, may suggest capacity for higher resiliency among older sexual minorities compared to their heterosexual counterparts. These findings are also consistent with social identity approaches to health that assume collectivist and creative strategies are often adopted among minority groups to confront the ill effects of discrimination on wellbeing (21, 22). Similarly, shared care is a form of social support, consistent with social identity approaches that posit that positive connections to one’s group can lead to positive wellbeing (59). Further research is needed to more directly investigate how opportunities for social bonding among older minorities may create a sense of belonging and, in turn, diminish aging-related challenges in late adulthood.

Importantly, suicidal ideation and attempts among baby boomer SGM populations is not unavoidable despite the documented prevalence of this phenomena among participants. Indeed, public health interventions are likely needed to target the reduction of suicidal thoughts, planning, and completion among these individuals. In particular, an increase in LGBTQ+ inclusive and accessible palliative care services is essential, as these services are designed to reduce chronic pain and improve quality of life in the face of long-term health conditions. It is well known that medical staff at palliative and end-of-life care facilities would benefit from additional cultural competency training regarding the mental health needs of these distinctive populations, including factors that may reduce suicidal ideation and attempts (29, 33); older sexual minorities may opt to take advantage of these services in lieu of health-harming behaviors (i.e., suicide) if they perceive them as LGBTQ+ friendly (76).

### Limitations and future directions

Future research should continue to investigate how social inequality faced by older LGBTQ+ populations shapes aging and life course trajectories. The use of retrospective data does contain some limitations. For example, the use of this data may introduce possible recall biases. However, as noted by feminist researchers, self-reported data rarely offers an objective portrait of reality; moreover, the retelling of past events via stories is always a socially constructed act and subjective process (79). In addition, although retrospective data provides some insight into how early life events shape the wellbeing of older sexual minorities, longitudinal quantitative data is also needed to answer causal questions about the effects of homophobic stressors on various later life outcomes (e.g., poverty, health, successful aging). Additional insight into potential cohort differences in the unfolding of sexual minorities lives is also warranted, although the current study did reveal that baby boomers and the silent generation may differentially experience aging-related challenges. For example, boomers reported facing economic marginalization, which is—in part—accounted for by facing higher rates of workplace and medical discrimination earlier in life, due to being “out” at higher rates during culturally turbulent times for sexual minorities. Similarly, boomers reported higher rates of plans for assisted suicide than their silent generation counterparts, which may be due to a history of medical negligence; thus, greater attention should be paid to the long-term effects of the HIV epidemic on baby boomers’ lives (69).

There are also some sampling limitations present. Because the sample is predominately white, the findings may not fully generalize to and/or be differentially experienced by older sexual minorities of color. For example, the finding that study participants prefer suicide as an end-of-life plan in lieu of staying in a long-term care facility is possibly a story of whiteness. It is well known that there are higher rates of religiosity among Black and Latina/e/o populations, and thus there is likely a moral opposition to suicide among these communities (80). In addition, sexual minorities of color may experience more heightened financial distress, due to racial discrimination in pay and being clustered in low prestige and poor paying occupations earlier in life (81). Earlier life disadvantages, consequently, likely put LGBQ individuals of color in even greater financially precarious circumstances in late adulthood. Consequently, the story of cumulative inequality is an intersectional one, as multiple forms of discrimination faced earlier in life may worsen and exacerbate aging-related challenges for those who are multiply marginalized. Accordingly, future studies should further consider how the life course experiences of older sexual minorities of color may differ from their white counterparts.

This study is also limited in its ability to fully account for cumulative inequality processes faced by older bisexual and transgender persons. There were very few bisexual participants in the study, but this population’s aging and life course experiences could be distinct from their monosexual counterparts. For example, financial distress is likely exacerbated for bisexual people in late adulthood, due to the fact that they are more likely to live below the poverty line earlier in life (82). Moreover, it is well known that bisexual individuals report less acceptance into LGBTQ+ communities throughout their lives, which may manifest as lower social support in late adulthood (82). Lower social support, in turn, may also result in even higher rates of suicidal ideation among older bisexual populations compared to monosexual (non-bisexual) individuals. Further, transgender people were omitted from this study, due to potentially qualitative differences in aging and life course experience; however, research should further examine cumulative inequality processes among this population. For example, there is already some evidence that older transgender people face distinctive challenges in late adulthood, including financial barriers to advance care planning, rampant discrimination in paid care settings, housing insecurity, and worse mental and physical health outcomes (83, 84). Further, due to lifelong exposure to transphobic discrimination in the workforce, heightened financial barriers experienced later in life may compromise transgender people’s ability to access and afford quality medical care (83, 86). Understanding the long-term consequences of early life discrimination on the aging trajectories of older bisexual and transgender people, thus, should remain a priority for LGBTQ+ aging researchers.

In this article, I argue that the effects of early life discriminatory events loom large, often impacting later life experiences for sexual minorities. In particular, I find that exposure to early life stressors is associated with economic marginalization, medical distrust, assisted suicide planning, and community-centered care in late adulthood. Numerous questions regarding cumulative inequality processes are ripe for further investigation, including how the timing and duration of discrimination impacts aging and life course trajectories (14). In addition, researchers should further consider the cumulative impacts of wage loss over time, including the potential for delayed retirement, inability to secure affordable housing, or decreased financial access to a formal caregiver. Moreover, researchers should further investigate whether medical mistrust and corresponding rejection of stays in long-term care facilities lead to unmet medical needs, worsened physical health outcomes, and decreased life expectancy among LGBTQ+ populations. Questions regarding the ways in which discriminatory events cascade and trigger subsequent disadvantages across the life course must remain a priority for LGBTQ+ health and aging researchers. Finally, it is vital for public health scholars to further consider whether and how communal assets (e.g., chosen family, shared care, social support, religious networks) that sexual minority populations possess may help buffer negative effects of early life discrimination on late life experiences.

## Data Availability

The raw data supporting the conclusions of this article will be made available by the authors, without undue reservation.

## References

[1] Choi SK Meyer IH. LGBT Aging: A Review of Research Findings. Los Angeles, CA: The Williams Institute (2016). Available online at: https://williamsinstitute.law.ucla.edu/wp-content/uploads/LGBT-Aging-Aug-2016.pdf (Accessed June 30, 2026)

[2] Fredriksen-Goldsen KI. The future of LGBT+ aging: a blueprint for action in services, policies, and research. Generations. (2016) 40:6–15. PMC5375167 28366980

[3] Fredriksen-Goldsen KI. Resilience and disparities among lesbian, gay, bisexual, and transgender older adults. Public Policy Aging Rep. (2011) 21:3–7. doi: 10.1093/ppar/21.3.3, 26755892 PMC4706747

[4] Kimmel DC. Issues to consider in studies of midlife and older sexual minorities. In: Herdt G Vries B De, editors. Gay and Lesbian Aging: Research and Future Directions. Princeton, NJ: Springer; (2004). p. 3–28.

[5] Lottman R King A. Who can I turn to? Social networks and the housing, care, and support preferences of older lesbian and gay people in the UK. Sexualities. (2022) 25:9–24. doi: 10.1177/1363460720944588

[6] Dannefer D. Cumulative advantage/disadvantage and the life course: cross-fertilizing age and social science theory. J Gerontol B Psychol Sci Soc Sci. (2003) 58:S327–37. doi: 10.1093/geronb/58.6.S327 14614120

[7] Ferraro KF Shippee TP. Aging and cumulative inequality: how does inequality get under the skin? Gerontologist. (2009) 49:333–43. doi: 10.1093/geront/gnp034, 19377044 PMC2721665

[8] Hatch SL. Conceptualizing and identifying cumulative adversity and protective resources: implications for understanding health inequalities. J Gerontol B Psychol Sci Soc Sci. (2005) 50:130–4. doi: 10.1093/geronb/60.special_issue_2.s130, 16251584

[9] Crystal S Shea DG Reyes AM. Cumulative advantage, cumulative disadvantage, and evolving patterns of late-life inequality. Gerontologist. (2017) 57:910–20. doi: 10.1093/geront/gnw056, 27030008 PMC5881660

[10] O’Rand AM. The precious and precocious: understanding cumulative disadvantage and cumulative advantage of the life course. Gerontologist. (1996) 36:230–8. doi: 10.1093/geront/36.2.230, 8920094

[11] Choi E Tang F Copeland VC. Racial/ethnic inequality among older works: focusing on whites, blacks, and Latinos within the cumulative advantage/disadvantage framework. J Soc Serv Res. (2017) 43:18–36. doi: 10.1080/01488376.2016.1235068

[12] Kravitz-Wirts N. Cumulative effects of growing up in separate and unequal neighborhoods on racial disparities in self-rated health in early adulthood. J Health Social Behav. (2016) 57:453–70. doi: 10.1177/0022146516671568, 27799591 PMC5463536

[13] Wallace S Nazroo J Becares L. Cumulative effect of racial discrimination on the mental health of ethnic minorities in the United Kingdom. Am J Public Health. (2016) 106:1294–300. doi: 10.2105/AJPH.2016.303121, 27077347 PMC4984732

[14] Fredriksen-Goldsen KI Bryab AEB Jen S Goldsen J Kim H-J Muraco A. The unfolding of LGBT lives: key events associated with health and well-being in later life. Gerontologist. (2017) 57:S15–29. doi: 10.1093/geront/gnw185, 28087792 PMC5241757

[15] Meyer IH Russell ST Hammack P Frost DM Wilson BDM. Minority stress, distress, and suicide attempts in three cohorts of sexual minority adults: a U.S. probability sample. PLoS One. (2021) 16:e0246827. doi: 10.1371/journal.pone.0246827, 33657122 PMC7928455

[16] Russell ST Mallory AB Fish JN Frost DM Hammack PL Lightfoot M . Distribution and prevalence of health in a national probability sample of three cohorts of sexual minority adults in the United States. LGBT Health. (2022) 9:564–70. doi: 10.1089/lgbt.2020.0505, 35856801 PMC9734014

[17] Eppler-Hattab R Steindorsdottier BD. The working life course of aging LGBTQ workers: an intersectional perspective and a theoretical framework. Work Aging Retir. (2025):waaf020. doi: 10.1093/workar/waaf020

[18] Badgett MVL. The Economic Case for LGBT Equality. Boston, MA: Beacon Press (2020).

[19] Robinson-Wood T Weber A. "Deconstructing multiple oppressions among LGBT older adults". In: Harley DA Teaster PB, editors. Handbook of LGBT Elders. Princeton, NJ: Springer (2016). p. 65–81.

[20] Torres S Lacy G. "Life course transitions, personal networks, and social support for LGBT+ elders: implications for physical and mental health". In: LeBlanc AJ Perry BL, editors. Sexual and Gender Minority Health: Advances in Medical Sociology. Leeds, England: Emerald Publishing Limited (2021). p. 157–79.

[21] Haslam SA Jetten J Postmes T Haslam C. Social identity, health, and well-being: an emerging agenda for applied psychology. Appl Psychol. (2009) 58:1–23. doi: 10.1111/j.1464-0597.2008.00379

[22] Jetten J Haslam SA Cruwys T Greenaway KH Haslam C Steffens NK. Advancing the social identity approach to health and well-being: progressing the social cure research agenda. Eur J Soc Psychol. (2017) 47:789–802. doi: 10.1002/ejsp.2333

[23] Hatzenbuehler ML. How does sexual minority stigma “get under the skin”? A psychological mediation framework. Psychol Bull. (2009) 135:707–30. doi: 10.1037/a0016441, 19702379 PMC2789474

[24] Meyer IH. Minority stress and mental health in gay men. J Health Soc Behav. (1995) 36:38–56. doi: 10.2307/2137286 7738327

[25] Frost DM Meyer IH Lin A Wilson BDM Lightfoot M Russell ST . Social change and the health of sexual minority individuals: do the effects of minority stress and community connectedness vary by age cohort. Arch Sex Behav. (2022) 51:2299–316. doi: 10.1007/s10508-022-02288-6, 35411489 PMC9192485

[26] Miller LR Tabor JA. A life course perspective on sexual minorities: implications for the study of homophobic prejudice and discrimination. Sociol Compass. (2024) 18:e13207. doi: 10.1111/soc4.13207

[27] Fredriksen-Goldsen KI Kim H-J Bryan AEB Shiu C Emlet CA. The cascading effects of marginalization and pathways of resilience in attaining good health among LGBT older adults. Gerontologist. (2017) 57:S72–83. doi: 10.1093/geront/gnw170, 28087797 PMC5241752

[28] Fredriksen-Goldsen KI Hoy-Ellis CP Muraco A Goldsen J Kim H-J. "The health and well-being of LGBT older adults: disparities, risks, and resilience across the life course". In: Orel NA Fruhauf CA, editors. The Lives of LGBT Older Adults: Understanding Challenges and Resilience. Washington, DC: American Psychological Association

[29] Lampe NM Barbee H Tran NM Bastow S McKay T. Health disparities among lesbian, gay, bisexual, transgender, and queer older adults: a structural competency approach. Int J Aging Hum Dev. (2024) 98:39–55. doi: 10.1177/0091415023117183 PMC10598237 37122150

[30] Annele U Satu KJ Timo ES. Definitions of successful aging: a brief review of multidimensional concept. Acta Biomed. (2019) 90:359–63. doi: 10.23750/abm.v90i2.8376 PMC6776218 31125022

[31] Rowe JW Kahn RL. Successful aging. Gerontologist. (1997) 37:433–40. doi: 10.1093/geront/37.4.433, 9279031

[32] Rowe JW Kahn RL. Successful aging 2.0: conceptual expansions for the 21st century. J Gerontol B. (2015) 70:593–6. doi: 10.1093/geronb/gbv025, 25878054

[33] Miller LR. Queer aging: older lesbian, gay, and bisexual adults’ visions of late life. Innov Aging. (2023) 7:1–9. doi: 10.1093/geroni/igad021, 37063703 PMC10101047

[34] Weston K. Families We Choose: Lesbians, Gays, Kinship. New York, NY: Columbia University Press (1997).

[35] Wilson K Kortes-Miller K Stinchcombe A. Staying out of the closet: LGBT older adults’ hopes and fears in considering end-of-life. Canad J Aging = La revue canadienne du vieillissement. (2018) 37:22–31. doi: 10.1017/S0714980817000514, 29335034

[36] Lampe NM. Satisficing death: ageing and end-of-life preparation among transgender older Americans. Sociol Health Illn. (2024) 46:887–906. doi: 10.1111/1467-9566.13741, 38149851 PMC11189757

[37] Hughes M Cartwright C. Lesbian, gay, bisexual, and transgender people’s attitudes to end-of-life decision-making and advance care planning. Australas J Ageing S205; 34: 39–43. doi: 10.1111/ajag.12268 26525446

[38] Seelman KL Lewinson T Engleman L Allen A. Motivations for advance care and end-of-life planning among lesbian, gay, and bisexual older adults. Qual Soc Work. (2019) 18:1002–16. doi: 10.1177/1473325018792396, 33343234 PMC7747803

[39] Thomeer MB Donnell R Reczek C Umberson D. Planning for future care and the end of life: a qualitative analysis of gay, lesbian, and heterosexual couples. J Health Soc Behav. (2017) 58:473–87. doi: 10.1177/0022146517735524, 29172768 PMC5718053

[40] Flatt JD Cicero EC Kittle KR Brennan-Ing M Anderson JG Wharton W . Advancing gerontological health research with sexual and gender minorities across the globe. J Gerontol Nurs. (2022) 48:13–20. doi: 10.3928/00989134-20220304-03, 35343840 PMC9949509

[41] Caceres BA Brody A Luscombe RE Primiano JE Marusca P Sitts EM . A systematic review of cardiovascular disease in sexual minorities. Am J Public Health. (2017) 107:e13–21. doi: 10.2105/AJPH.2016.303630, 28207331 PMC5343694

[42] Goldsen-Fredriksen KI Kim H-J Shui C Bryan AEB. Chronic health conditions and key health indicators among lesbian, gay, and bisexual older adults, 2013-2014. Am J Public Health. (2017) 107:1332–8. doi: 10.2105/AJPH.2017.303922 PMC5508186 28700299

[43] Butler ES McGlinchey E Juster R-P. Sexual and gender minority sleep: a narrative review and suggestions for future research. J Sleep Res. (2019) 29:e12928. doi: 10.1111/jsr.12928, 31626363

[44] Akre ER Miller KEM McKay T. Variation in subjective cognitive decline among older adults in the US south based on sexual orientation and gender identity. Research on Aging (2026). doi: 10.1177/0164027526142997, 41789507

[45] Hsieh N Liu H Lai W-H. Elevated risk of cognitive impairment among older sexual minorities: do health conditions, health behaviors, and social connections matter? Gerontologist. (2021) 61:352–62. doi: 10.1093/geront/gnaa136 PMC8023357 32951038

[46] Romanelli RJ Rosenblatt AS Marcum ZA Flatt JD. Cognitive impairment in sexual and gender minority groups: a scoping review of the literature. LGBT Health (2023) 11:178–92. doi: 10.1089/lgbt.2023.0095, 37824757

[47] Fredriksen-Goldsen KI Kim H-J Shiu C Goldsen J Emlet CA. Successful aging among LGBT older adults: physical and mental health-related quality of life by age group. Gerontologist. (2015) 55:154–68. doi: 10.1093/geront/gnu081, 25213483 PMC4542897

[48] Hyun-Jun K Fredriksen-Goldsen KI. Living arrangement and loneliness among lesbian, gay, and bisexual older adults. Gerontologist. (2016) 56:548–58. doi: 10.1093/gerontgnu083 PMC4873763 25213482

[49] Perone AK Ingersoll-Dayton B Watkins-Dukhie K. Social isolation loneliness among LGBT older adults: lessons learned from a pilot friendly caller program. Clin Soc Work J. (2020) 48:126–39. doi: 10.1007/s10615-019-00738-8

[50] Kittle K Boerner K Kim K. The role of minority stress and social resources in the healthcare utilization of aging LGBT adults. Innov Aging. (2021) 5:173–3. doi: 10.1093/geroni/igab046.660

[51] Hicks M Conner NE. Resilient ageing: a concept analysis. J Adv Nursin. (2014) 70:744–55. doi: 10.1111/jan.12226, 23919385

[52] Pereira H Banerjee D. Successful aging among older LGBTQIA+ people: future research and implications. Front Psych. (2021) 12:756649. doi: 10.3389/fpsyt.2021.756649, 34759850 PMC8573324

[53] Friend RA. A theory of successful aging. J Homosex. (1991) 20:99–118. doi: 10.1300/J082v20n03_07 2086655

[54] MetLife Mature Market Institute Still Out, Still Aging: The MetLife Study of Lesbian, Gay, Bisexual, and Transgender Baby Boomers New York, NY The MetLife Market Institute (2010). Available online at: https://www.sageusa.org/wp-content/uploads/2025/08/sageusa-still-out-still-aging.pdf

[55] shuster s Westbrook L. Reducing the joy deficit in sociology: a study of transgender joy. Soc Probl. (2014) 71:791–804. doi: 10.1093/socpro/spac034

[56] Calasanti T de Vries B Geng J. Gay and lesbian older adults caring for their spouses living with dementia: positive identity and community at the margins. Res Aging. (2026). doi: 10.1177/01640275261435492 41848186

[57] Seery MD Holman EA Silver RC. Whatever does not kill us: cumulative lifetime adversity, vulnerability, and resilience (2010) 99:1025–41. doi: 10.1037/a0021344, 20939649

[58] Schafer MH Shippee TP Ferraro KF. When does disadvantage not accumulate? Toward a sociological conceptualization of resilience. Schweiz Z Soziol. (2014) 35:231–51. PMC3920188 24526803

[59] Wakefield JRH Bowe M Kellezi B McNamara N Stevenson C. When groups help and when groups harm: origins, developments, and future directions of the “social cure” perspective of group dynamics. Soc Personal Psychol Compass. (2019) 13:e12440. doi: 10.1111/spc3.12440

[60] Reczek C. Queering Aging Onondaga County, New York Syracuse University, Aging Studies Institute (2018). Available online at: https://asi.syr.edu/research/publications/queering-aging/ (Accessed June 30, 2026)

[61] Atkinson R. The Life Story Interview. Thousand Oaks, CA: Sage (1998).

[62] Ramirez-Valles J. Queer Aging: The Gayby Boomers and the New frontier for Gerontology. Oxford, UK: Oxford University Press (2016).

[63] Stone AL. The geography of research on LGBTQ life: why sociologists should study the south, rural queers, and ordinary cities. Sociol Compass. (2018) 12:312638. doi: 10.1111/soc4.12638

[64] Charmaz K. "Qualitative interviewing and grounded theory analysis". In: Gubrium JF Holstein JA, editors. Handbook of Interview Research. Thousand Oaks, CA: Sage (2002). p. 671–5.

[65] Emerson RM Fretz RI Shaw LL. Writing Ethnographic Fieldnotes. Chicago, IL: University of Chicago Press (1995).

[66] Cohen DJ Crabtree BF. Evaluative criteria for qualitative research in healthcare: controversies and recommendations. Ann Family Med. (2008) 6:331–9. doi: 10.1370/afm.818, 18626033 PMC2478498

[67] Capistrant BD Nakash O. Suicide risk for sexual minorities in middle and older age: evidence from the national survey on drug use and health. Am J Geriatr Psychiatry. (2017) 27:559–63. doi: 10.1016/j.jagp.2018.12.023, 30770188

[68] Chan AS Po Yee Lo I Yan E. Health and social inclusion: the impact of psychological well-being and suicide attempts among older men who have sex with men. Am J Mens Health. (2022) 16:1–9. doi: 10.1177/15579883221120985 PMC9465597 36082415

[69] Rosenfeld D Bartlam B Smith RD. Out of the closet and into the trenches: gay male baby boomers, aging, and HIV/AIDS. Gerontologist. (2012) 52:255–64. doi: 10.1093/geront/gnr138, 22298746

[70] Martinson M Berridge C. Successful aging and its discontents: a systematic review of the social gerontology literature (2015) 55:58–69. doi: 10.1093/geront/gnu037, PMC4986586 24814830

[71] Plasencia MZ. I don’t have much money, but I have a lot of friends: how poor older Latinxs find social support in peer friendship networks (2023) 70:755–72. doi: 10.1093/socpro/spab081,

[72] Golant SM. The distance to death perceptions of older adults explain why they age in place: a theoretical examination. J Aging Stud. (2020) 54:100863. doi: 10.1016/j.jaging.2020.100863, 32972627 PMC7489887

[73] Sandberg LJ Marshall BL. Queering aging futures. Societies. (2017) 7:21. doi: 10.3390/soc7030021

[74] Mohatt NV Thompson AB Thai ND Tebes JK. Historical trauma as public narrative: a conceptual review of how history impacts present-day health. Soc Sci Med. (2014) 106:128–36. doi: 10.1016/j.socscimed.2014.01.043, 24561774 PMC4001826

[75] Shukla M Schilt-Solberg M Gibson-Scipio W. Medical mistrust: a concept analysis. Nurs Rep. (2025) 15:103. doi: 10.3390/nursrep15030103, 40137676 PMC11944586

[76] Conrad KJ Goldberg SK O’Neill K. Religiosity among LGBT Adults in the US. Los Angelas, CA: The Williams Institute. Available online at: https://williamsinstitute.law.ucla.edu/wp-content/uploads/LGBT-Religiosity-Oct-2020.pdf (Accessed June 30, 2026)

[77] Clark KD Luong S Lunn MR Flowers E Bahalkeh E Lubensky ME . Healthcare mistreatment, state-level policy protections, and healthcare avoidance among gender minority people. Sex Res Social Policy. (2022) 19:1717–30. doi: 10.1007/s13178-022-00748-1339 PMC9701649 36458212

[78] Kcomt L Gorey KM Barrett BJ McCabe SE. Healthcare avoidance due to anticipated discrimination among transgender people: a call to create trans-affirmative environments. SSM Popul Health. (2020) 11:1006808. doi: 10.1016/j.ssmph.2020;100608 PMC7276492 32529022

[79] DeVault ML Gross G. "Feminist qualitative interviewing: experience, talk, and knowledge". In: Hesse-Biber SN, editor. The Handbook of Feminist Research: Theory and Praxis. Thousand Oaks, CA: Sage (2022). p. 206–36.

[80] Harley DA. "African-American and Black LGBT elders". In: Harley DA Teaster PB, editors. Handbook of LGBT Elders: An Interdisciplinary Approach to Principles, Practices, and Policies. New York, NY: Springer (2015). p. 105–34.

[81] Fredriksen-Goldsen KI Shiu C Bryan AEB Goldsen J Kim HJ. Health equity and aging of bisexual older adults: pathways of risk and resilience. J Gerontol B Psychol Sci Soc Sci. (2017) 72:468–78. doi: 10.1093/geronb/gbw120, 27815302 PMC5927101

[82] Catlett L Acquaviva KD Campbell L Ducar D Page EH Patton J . End-of-life care for transgender older adults. Global Qualit Nurs Res. (2023) 10:1–13. doi: 10.1177/23333936231161128, 36992796 PMC10041615

[83] Witten TM. End of life, chronic illness, and trans-identities. J Soc Work End Life Palliat Care. (2014) 10:34–58. doi: 10.1080/15524256.2013.877864, 24628141

[84] Catlett L Simmons A Williams IC . Multilevel Factors Affecting Health Equity for Transgender and Gender Diverse Older Adults. Qualitative Health Research. (2025). doi: 10.1177/10497323251342162, 40497716 PMC13672935

